# Rare vascular anomaly mimicking bronchogenic carcinoma

**DOI:** 10.4103/0970-2113.80336

**Published:** 2011

**Authors:** Sanjeev Kumar Verma, Vineet Mahajan

**Affiliations:** *Department of Pulmonary Medicine, Chhatrpati Shahuji Maharaj Medical University, Lucknow, Uttar Pradesh, India*; 1 *Department of Pulmonary and Critical Care, Dayanand Medical College & Hospital, Ludhiana, Punjab, India*

**Keywords:** Pulmonary artery sling, bronchogenic carcinoma, lung anomaly

## Abstract

We report a case of anomalous left pulmonary artery (pulmonary artery sling) detected incidentally on computed tomography thorax. This was carried out to rule out bronchogenic carcinoma in a patient of chronic obstructive pulmonary disease who presented with streaking. He was a chronic smoker having bilateral hilar prominence on chest radiograph.

## INTRODUCTION

Anomalous left pulmonary artery (pulmonary artery sling) is a developmental abnormality in which the left pulmonary artery arises from the right pulmonary artery and enters the left hilum after passing between the trachea and the oesophagus. It usually occurs in neonates and young infants, is often associated with tracheobronchial or cardiovascular anomalies, and is almost always fatal if untreated.[1] If not detected in early life, it may present as a mediastinal abnormality in later life. We are reporting such a case which was detected in an elderly patient.

## CASE REPORT

**Figure 1:**
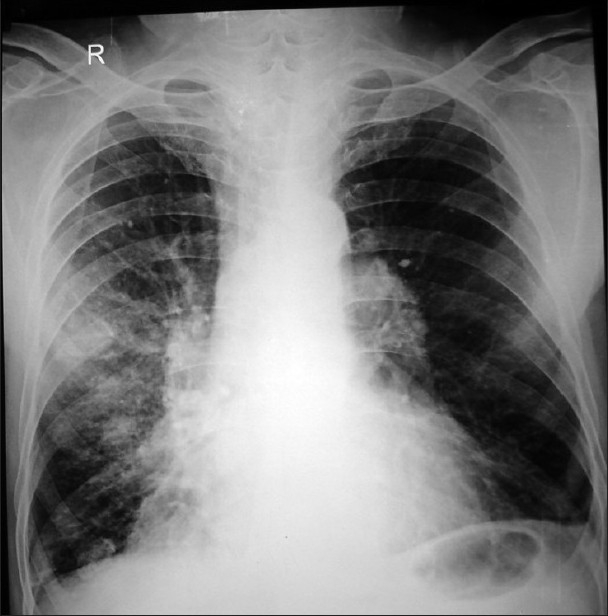
Chest radiograph showing bilateral hilar prominence

**Figure 2:**
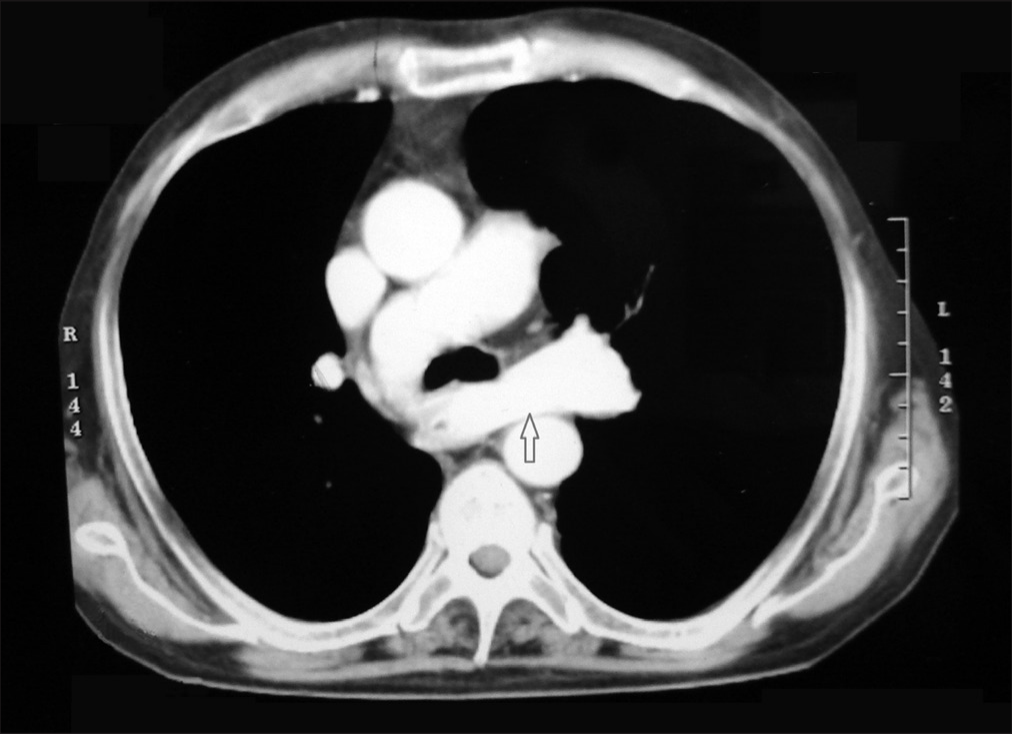
Section of CT thorax showing aberrant course of left pulmonary artery (arrow)

**Figure 3:**
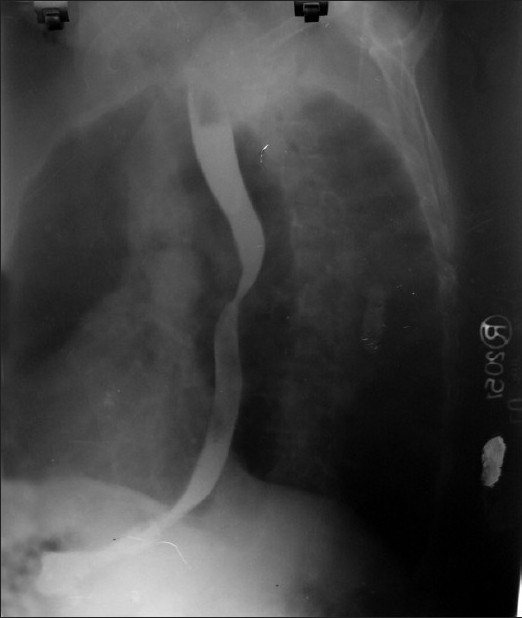
Radiograph of barium study showing anterior indentation of oesophagus

A 56-year-old man was admitted to our department with complaints of severe respiratory distress, wheeze, cyanosis, and streaking. The patient, farmer by occupation, was known case of chronic obstructive pulmonary disease from last 15 years. He was on inhaled medications, but was noncompliant. He was a chronic smoker with pack-year 40. On physical examination, jugular venous pressure was raised and he had bilateral pitting pedal edema. On inspection, he had hyperinflated chest and bilateral, diffuse rhonchi were present on auscultation. Arterial blood gas done on admission was suggestive of respiratory acidosis with metabolic compensation. Therefore, a diagnosis of cor pulmonale with acute exacerbation of chronic obstructive pulmonary disease and type II respiratory failure was made. Routine investigations were within normal limits. Chest radiograph had bilateral hilar prominence with signs of hyperinflation [Figure 1]. Patient was given bronchodilators, diuretics, antibiotics, and noninvasive positive airway pressure with significant improvement in 24 h. As there was bilateral hilar prominence in a chronic smoker complaining of streaking, CT thorax was performed to rule out bronchogenic carcinoma. To our surprise, the hilar prominence was found to be an anomalous left pulmonary artery from the right pulmonary artery and coursing behind the carina to reach the left hilum [Figure 2]. Besides this, there were bilateral emphysematous bullae. On barium study, a smooth indentation in the anterior wall of middle one-third of oesophagus below the level of arch of aorta was seen [Figure 3]. Echocardiography was suggestive of right ventricular hypertrophy and pulmonary artery hypertension. Therefore, bilateral hilar prominence in a chronic smoker thought to be bronchogenic carcinoma came out to be an incidental rare pulmonary vascular anomaly.

## DISCUSSION

Pulmonary artery sling is created by the anomalous origin of the left pulmonary artery from the posterior aspect of the right pulmonary artery. The anomalous left pulmonary artery courses over the right main stem bronchus and then from right to left, posterior to the trachea or carina and anterior to the oesophagus, to reach the hilum of the left lung. This compresses the lower trachea and right main stem bronchus producing upper airway symptoms. Compression caused by the sling can produce obstructive emphysema and/or atelectasis of the right as well as the left lung.

Incidence of associated congenital malformations varies and has been noted to occur in 81% in one study[1] and primarily involves the tracheobronchial tree and cardiovascular system. Associated tracheobronchial abnormalities may occur, especially complete tracheal rings and tracheomalacia. Hypoplasia and stenosis of tracheal segments occur and can potentiate airway obstruction.[2] Congenital heart defects are found in 50% cases, most commonly atrial septal defect, patent ductus arteriosus, ventricular septal defect, and left superior vena cava. Gastrointestinal, genitourinary, vertebral, and thyroid anomalies have also been reported.[2]

Although it is most frequently diagnosed in symptomatic children, the condition can be found in asyptomatic adults as an incidental finding in whom it can mimic a mediastinal adenopathy.[3–5] A pulmonary sling is apparently a benign and asymptomatic condition in adults.[6]

As this is a rare defect, overall frequency is not known and there is no sexual or racial predominance. In one study, there has been shown somewhat male predominance. Specific etiology of the pulmonary artery sling is unknown. The pathogenesis of the anomaly has been hypothesized to be faulty development or reabsorption of the ventral portion of the left sixth aortic arch, leaving the developing left pulmonary plexus to connect with the right sixth aortic arch. There is evidence that it may be related to a 22q11.2 chromosomal deletion. It can be diagnosed on a simple chest radiograph. The lower trachea is deviated to the left and may appear compressed on its right side with hyperinflation in both lungs. Lateral view may demonstrate a density anterior to the oesophagus and posterior to the trachea just above the carina. The diagnostic procedure of choice is barium swallow. An anterior indentation of the oesophagus on the lateral projection is diagnostic of pulmonary artery sling. Echocardiography can show the course of the anomalous left pulmonary artery as well as any associated congenital heart defect. Bronchoscopy is generally not recommended. If performed, tracheal compression is noted and accompanying tracheomalacia and/or tracheal stenosis is seen. Currently, magnetic resonance imaging/magnetic resonance angiography and/or CT scan can be helpful in delineating the details of the anatomy. Spiral CT angiography is a one-stop shop that has the advantage of avoiding the invasive investigations such as bronchoscopy, tracheobronchography, and pulmonary artery angiography.[7]

As far as the treatment is concerned in infants, medical care is supportive until the patient can undergo definitive surgical correction. Hypoxemia and respiratory distress should be treated with supplemental oxygen and endotracheal intubation if indicated. Pneumonia should be treated with appropriate antibiotics. Infants without airway obstruction and with minimal symptoms may not require surgical intervention. This scenario, however, is the rare exception. Survival of symptomatic infants is unlikely without early surgical intervention. Surgical survivors may be free of significant symptoms at long-term follow-up but, because many demonstrate some persistent airway obstruction, they should be followed closely for both airway as well as pulmonary artery complications. On the other hand, as the adults are mostly asymptomatic, no active surgical intervention is required.
